# The Impact of the International Cooperation On Familial Hypercholesterolemia Screening and Treatment: Results from the ScreenPro FH Project

**DOI:** 10.1007/s11883-019-0797-3

**Published:** 2019-06-22

**Authors:** Richard Ceska, Gustavs Latkovskis, Marat V. Ezhov, Tomas Freiberger, Katarina Lalic, Olena Mitchenko, Gyorgy Paragh, Zaneta Petrulioniene, Belma Pojskic, Katarina Raslova, Aleksandr B. Shek, Branislav Vohnout, Tereza Altschmiedova, Veronika Todorovova

**Affiliations:** 1 Third Department of Medicine – Department of Endocrinology and Metabolism of the First Faculty of Medicine, Charles University and General University Hospital, Prague, Czech Republic; 2 Latvian Research Institute of Cardiology, Faculty of Medicine, University of Latvia, Riga, Latvia; 3 Paul Stradins Clinical University Hospital, Riga, Latvia; 4 National Cardiology Research Center, Moscow, Russia; 5 Centre for Cardiovascular Surgery and Transplantation, Brno, Czech Republic; 6 Medical Faculty, Masaryk University, Brno, Czech Republic; 7 Clinic for Endocrinology, Diabetes and Metabolic Diseases, Belgrade, Serbia; 8 Faculty of Medicine, University of Belgrade, Belgrade, Serbia; 9 National Registry Coordinator in Ukraine, Kiev, Ukraine; 10 Department of Internal Medicine, Faculty of Medicine, University of Debrecen, Debrecen, Hungary; 11 Vilnius University Faculty of Medicine, Vilnius, Lithuania; 12 Vilnius University Hospital Santaros Klinikos, Vilnius, Lithuania; 13 Cantonal Hospital Zenica, Zenica, Bosnia and Herzegovina; 14 Coordination Center for Familial Hyperlipidemias, Slovak Medical University, Bratislava, Slovakia; 15 Head of Department of Ischemic Heart Disease and Atherosclerosis, Republican Specialised Center of Cardiology, Tashkent, Uzbekistan; 16 Institute of Nutrition, Faculty of Nursing and Health Professional Studies and Coordination Centre for Familial Hyperlipoproteinemias, Slovak Medical University in Bratislava, Bratislava, Slovakia; 17 Institute of Epidemiology, School of Medicine, Comenius University, Bratislava, Slovakia

**Keywords:** Familial hypercholesterolemia, FH, ScreenPro FH, Evolocumab, Alirocumab, LDL-C

## Abstract

**Purpose of Review:**

Familial hypercholesterolemia (FH) is often perceived and described as underdiagnosed and undertreated, though effective treatment of FH is available. Owing to the mentioned facts, it is ever more imperative to screen and treat FH patients. Subsequent to the identification of patients, the project focuses on the improvement of their prognoses. The ScreenPro FH project was established as a functional international network for the diagnosis, screening, and treatment of FH. Individual countries were assigned goals, e.g., to define the actual situation and available treatment. With “central support,” more centers and countries participated in the project. Subsequently, individual countries reported the results at the beginning and end of the project. Collected data were statistically evaluated.

**Recent Findings:**

The increasing number of patients in databases, from 7500 in 2014 to 25,347 in 2018, demonstrates the improvement in overall effectiveness, as well as an increase in the number of centers from 70 to 252. Before all, LDL-C decreased by 41.5% and total cholesterol by 32.3%. As data from all countries and patients were not available at the time of the analysis, only those results from 10 countries and 5585 patients at the beginning of the project and at the time of writing are included.

**Summary:**

Our data are quite positive. However, our results have only limited validity. Our patients are far from the target levels of LDL-C. The situation can be improved with the introduction of new therapy, PCSK9-i, evolocumab, and alirocumab. International cooperation improved the screening of FH and finally led to an improvement in cardiovascular risk.

## Introduction

Familial hypercholesterolemia (FH) is still, despite great recent progress, underestimated, underdiagnosed, and undertreated, and it represents a significant problem as a common risk factor for the premature development of coronary heart disease (CHD) [1, 2••]. FH is a monogenic disease transmitted through autosomal dominant inheritance and stems from either an LDL-R defect, familial defective apolipoprotein B-100 (FDB), or PCSK9 gain-of-function mutations [3, 4•].

FH is an example of a disease that, by its very nature, allows us to study the relationship between lipid metabolism, especially LDL-cholesterol (LDL-C), and atherosclerosis, as well as the premature manifestation of cardiovascular disease (CVD) [1, 5]. FH occurs with a frequency of 1:250–1:500 and is one of the most common congenital metabolic disorders [6].

When we started to monitor and study FH, we primarily dealt with the mechanism of the development of the disease, and its genetic background and epidemiology [7–9]. As for the prognosis of patients, there was very little we could do. There were no options other than selecting the highest-risk patients with increased concentrations of lipoprotein/a/ (Lp/a/) [10] and trying to influence the most important risk factor beyond the scope of pharmacological treatment: the smoking of cigarettes [11]. However, the situation changed dramatically at the end of the 1980s, and especially in the 1990s, when statins were introduced to the market. Statins were widely used, and initially, their priority use was appropriate for the treatment of FH [12, 13]. In practice, treatment with statins has had an immediate impact as shown by a dramatic decrease in mortality, especially in FH patients under the age of 40 [14–16]. Later, the pharmacotherapy of FH was boosted by the introduction of ezetimibe [17, 18].

Though effective treatment was possible, interest in FH was relatively low; this situation led to numerous initiatives, both on the national and international level, such as the MedPed (Make Early Diagnosis in Medical Pedigree) project [19•], and the FH Foundation to name a few [20]. It should be noted that the Czech Republic, as the lead country of the described project, became one of the most successful countries in the identification of FH patients not only in Europe, but globally [21]. Although these activities were successful, most patients remained undiagnosed, were treated with low doses of medicaments, and did not receive the maximum therapy [22, 23]. In addition, even those patients cared for in specialized centers did not often reach the target values and LDL-C values in FH patients remained high above the upper limit of normal [24]. Of course, the cardiovascular (CV) risk also remained very high.

This is why both physician and patient communities involved in the field of FH appreciated the introduction of a novel generation of medicines into this field. Anti-PCSK9 monoclonal antibodies, also called the biological treatment for hypercholesterolemia or, simply, PCSK9-inhibitors (PCSK9-i), introduced the possibility of decreasing LDL-C levels by 40–60% when used additively to the maximum tolerated dose of lipid-lowering drugs [25]. They have been investigated in various populations, including FH [26]. Not only do they decrease the levels of LDL-C, but also Lp/a/ which is another independent risk factor for CV diseases [27, 28]. However, the most important fact in support of the use of these medicines in the treatment of FH is the results of randomized clinical studies in tens of thousands of enrolled patients with evolocumab (Fourier) [29, 30•], as well as those of alirocumab (Odyssey Outcomes) [31•], which show a reduction in CV event occurrence in a remarkably short time and, in one sub-analysis, even a decrease of overall mortality.

Also, the other medications for LDL-C lowering are in development, e.g., bempedoic acid and others [32]. It means that potent and powerful therapy becomes available for FH patients. The identification of FH patients and other highest-risk patients subsequently became one of the priorities of current preventive cardiology and clinical lipidology.

## Aim

Clearly, the aim of the project is to improve the identification, diagnostics, and treatment of FH patients in the regions of Central, Eastern, and Southern Europe (CESE). The standards of care for FH patients, as well as the identification of probands and affected members of families, greatly vary from country to country in the region. The awareness of FH among both experts and the general population varies as well. Therefore, when building the lipid center network and performing educational activities, exploiting the knowledge of more successful countries is a deciding factor. The ultimate objective of the project is the improvement of the lipid profile, the total CV risk and, finally, the improvement of the patient prognosis.

## Methods

The ScreenPro FH Project is an international project dedicated to the improvement of complex care—screening, diagnosis, and treatment of FH in CESE. Originating in seven countries, it allowed us to identify enthusiastic country leaders and create national and international networks of lipid centers coordinated by the project leaders. Individual countries were then set goals, the first of which was to define the actual situation and to determine the available treatment. From this point on, the project leaders regularly provided each country with information and instructions sent electronically or introduced during business meetings held in conjunction with major international congresses. With such “central support” (materials, education, web-based information), more and more centers and countries participated in the project.

Upon completion of the three-year project, each country reported the baseline results (from the beginning of the project) and results after the inclusion of the patients to the national database. Nowadays, the basic lipid parameters are available.

Data from individual countries included the number of FH patients, and averages and standard deviations of lipid parameters from the beginning of the project and after the inclusion of the patients to the national database. Average values of lipid parameters of individual countries were summed in relation to the number of patients, and the difference of the given lipid parameter values was evaluated. The results were evaluated using STATISTICA 13 software. All conducted tests were both-sided. The established level of significance was *α* = 0.05 in all tests. It must also be stressed that all the data from participating countries are aggregate data, not individual patient data.

### Description of the Situation in Countries

The countries involved in the ScreenPro FH project comprise about 500 million inhabitants in total. If we consider the prevalence of FH 1:250–500 [33], it constitutes approximately 1–2 million people suffering from this genetic disease. Although up-to-date results and analyses support the theory that the occurrence of FH in the overall population is 1:250, the participating countries have long estimated a prevalence of 1:500. The actual number of FH patients is, therefore, much higher in each individual country, and the rate of diagnosed cases is, on the contrary, much lower.

#### Bosnia and Herzegovina

Approximately 7000 people, in a country of 3.5 million, suffer from FH. Other than the National Centre in Zenica, there are two more centers operating in Bosnia and Herzegovina. The number of diagnosed FH patients, or, rather, FH patients registered in the database, is 1500; there is neither a lipid network nor patient organization in the country. Potential patients are selected from hospital databases based on an LDL-C level higher than 5 mmol/L. Diagnostics is based on the Dutch Lipid Clinic Network Criteria (DLCNC). The only treatment available to doctors is statins, with no option of combining them with ezetimibe or PCSK9-i. LDL apheresis is not an option in Bosnia and Herzegovina either. The FH program is focused on educational activities for general practitioners for children and adults, internists, and ophthalmologists.

#### Bulgaria

Bulgaria has 7.2 million inhabitants and the estimated number of FH patients is 14,000. The database compiles data from the national center, as well as from six other centers, and consists of 220 patients. The FH program commences at Intensive Cardiac Care Units, i.e., using patients with previous case histories of CVD. Diagnostics is based on the DLCNC. Therapy is based on statins which can be combined with ezetimibe, and upon achieving six or more points, based on the DLCNC, patients can also receive PCSK9-i therapy.

#### Croatia

In a country of 4.2 million inhabitants, the estimated number of FH patients is approximately 8500 with 150 patients included in the database. Croatia boasts a national center at the University Hospital in Zagreb, and four more centers are being planned. The existing lipid network is based on the MedPed project which is also the basis of the National MedPed program. The DLCNC are applied for the diagnostics of the disease, with treatment options including not only statins and ezetimibe but also PCSK9-i or LDL apheresis. Target LDL-C levels are based on the available recommended methods. No patient organization has been established in the country.

#### Czech Republic

The Czech Republic has 10.5 million inhabitants; thus, the estimated number of potential FH patients is more than 21,000. The country has a rich network of 69 centers including national centers in Prague at the General Faculty Hospital (VFN) and in Brno at St. Anne’s Hospital. These centers are already cooperating with more than 8000 patients, and the FH program supported by the Czech Society for Atherosclerosis cooperates closely with this network of lipid centers. Coordinators help physicians to operate the centers and to enter patients into the database. Diagnostics is based on the modified MedPed criteria with genetic testing available. Statins, ezetimibe, and PCSK9-i are available for treatment. Two centers offer also LDL apheresis. A patient organization was established and operates in the country.

#### Georgia

There is one center in Georgia, however, whose number of registered patients is unknown. In this country of 3.7 million inhabitants, the estimated number of FH patients is 7500.

#### Greece

Greece has almost 11 million inhabitants, and this corresponds to an estimated 21,000 FH patients. More than 600 patients have been diagnosed and are included in the database. The national center at the University Hospital in Ioannina cooperates with eight other centers. The FH program is based on a functioning network of lipid centers contributing to the national Hellas FH register. The DLCNC are used in diagnostics, and treatment options include statins, ezetimibe, and PCSK9-i. Four centers also perform LDL apheresis. A patient organization is also available for patients.

#### Hungary

In this country of 9.8 million inhabitants, the estimated occurrence of FH patients is 20,000 with 300 patients having been integrated into the ScreenPro FH database and monitored by two national centers in Debrecen and Budapest. Genetic analyses, sponsored by scientific grants, are also performed there, as well as in 18 regional centers. Hungary can be considered a country with a functioning lipid network. FH is diagnosed based on the DLCNC, and all the treatment modalities—statins, ezetimibe, PCSK9-i, and LDL apheresis—are available; the target levels of LDL-C are 1.8 mmol/L. An umbrella patient organization was also created for patients.

#### Kazakhstan

The Republic of Kazakhstan has 18.5 million inhabitants. The potential number of FH patients can thus be up to 40,000. Nevertheless, there is no information available on the number of patients included in the database.

#### Kyrgyzstan

In Kyrgyzstan, with a population of 5.8 million inhabitants, the occurrence of FH patients is estimated to be 11,700; these patients can be monitored in the national center in Bishkek or in 19 regional centers. The FH program deals with the analysis of the FH prevalence in patients with a premature manifestation of CVD, metabolic syndrome, and subsequent primary or secondary prophylaxis. Three hundred one patients, with diagnoses based on the DLCNC, are included in the database. Treatment options include statins, and LDL apheresis is also available. No patient organization has been founded yet.

#### Latvia

With a population of almost 2 million inhabitants, the occurrence of FH patients in Latvia is estimated to be approximately 4000, with an aggregate summary of data on 249 patients reported in the ScreenPro FH registry by the end of February 2019. Patients are monitored in the national center in Riga within the frames of the Latvian Registry of FH that was established in 2015 [34]. The registry currently is not financed by the government or any other organization, but it has effectively improved detection of cases from < 0.2% in early 2015 to more than 3% in early 2019. Index cases are diagnosed based on the DLCNC. The cascade screening is performed, and relatives are diagnosed based on 95th percentile of LDL-C. Statins, ezetimibe, and PCSK9-i are available, but only statins are 50% reimbursed for FH. LDL apheresis is not performed in this country. There is a working patient organization “ParSirdi.lv.”

#### Lebanon

Lebanon’s anticipated rate of FH incidence is 25 times higher than in Europe; i.e., in a land of 7.8 million inhabitants, the estimated occurrence of FH patients is 15,500, with only 38 patients included in the database. The higher incidence can be explained by the so-called founder effect and by the high number of marriages between blood relatives. A phenomenon called the Lebanese allele was described: qualifying FH in up to 81.5% of examined probands [35, 36]. There is one functioning national center in the country developing the FH program. Patients can be offered treatment with statins or ezetimibe; LDL apheresis is not available in this country, and no patient organization has been founded yet.

#### Lithuania

In the Lithuanian population of 2.9 million inhabitants, the occurrence of approximately 6000 FH patients is projected, with less than one-third of these patients included in the ScreenPro FH project. The national center was found in the capital, Vilnius, and four regional centers are being built. The Lithuanian High Cardiovascular Risk Primary Prevention Program (LiTHiR), started in 2006 and covered by health system, is the base for the functioning lipid network in the country. More than 250,000 middle-aged adults are screened every year and receiving primary prophylaxis. Data of > 92,000 individuals is currently included in the electronic database for detailed analysis. The prevalence of any dyslipidemia (DLP) among these patients is estimated to be 89%, and the prevalence of any type of severe DLP is 13.4%. The occurrence of patients with LDL-C levels ≥ 6 mmol/l in screened population is 3.2%, and in the subgroup of severe DLP, 24%. FH is diagnosed based on DLCNC. As far as treatment is concerned, patients can be offered statins, ezetimibe, and PCSK9-i, as well as LDL apheresis which is performed in one center. Genetic testing is available. The target is to achieve LDL-C levels in accordance with current European recommendations for the management of DLP treatment. Patient organization is being built under the umbrella of Lithuanian Heart Association.

#### Oman

Oman has a population of 5.2 million inhabitants, and the occurrence of FH patients is estimated to be approximately 10 thousand. Thirty-eight patients are included in the database.

#### Poland

Poland has a population of 38 million inhabitants, and 76,000 FH patients are to be expected, whereas fewer than 2000 have been diagnosed. Two national centers and seven other centers contribute to a functioning lipid network. The Polish national FH program is based on complex care for patients suffering from lipid metabolism disorders; diagnostics uses the DLCNC or Simon Broom Criteria. The program is focused on the selection of high-risk patients with the use of cascade screening in families with the option of genetic testing also available.

#### Romania

Almost 20 million people live in Romania, and taking into account the rates of occurrence we considered, up to 40,000 FH patients are assumed. The actual number of diagnosed patients is 69. The CardioPrevent Foundation Timisoara is the national center, and no other centers have been founded yet. No lipid network exists. Diagnostics is based on the DLCNC. Statins are available for treatment which can be combined with ezetimibe or PCSK9-i. LDL apheresis is not available. Target LDL-C levels depend on the degree of CV risk. No patient organization has been founded yet.

#### Russia

In a population of almost 147 million inhabitants, up to 300,000 patients suffering from FH are expected and 1400 FH patients have been successfully introduced to the database. The national Cardiology Research Center operates in Moscow, and 27 additional centers contribute to the lipid network. FH is normally diagnosed based on the DLCNC with DNA diagnostics also available. Treatment options available in Russia include statins and ezetimibe, as well as PCSK9-i; 10 centers also perform LDL apheresis. A patient organization is working in the country.

#### Serbia

Serbia has approximately 7.2 million inhabitants; thus more than 14,000 FH patients can be expected. Nine hundred of them have been included in the database. No specific FH program is available, there are eight regional centers and one national center functioning in the country with centralized screening, diagnostics, and treatment. Patients are referred to this site primarily by general practitioners. The DLCNC are used for diagnostics while statins, ezetimibe, and PCSK9-i are used for treatment, with LDL apheresis being available.

#### Slovakia

With a population of 5.4 million inhabitants, we expect up to 11,000 FH patients in Slovakia and more than 2500 of them are already included in the database. An extensive lipid network is established in the country; apart from the national center in Bratislava, there are 26 other centers (6 centers for pediatric patients). FH diagnostics can be performed based on the DLCNC, the Simon Broom Criteria, or the MedPed Criteria which is also the base of the Slovak FH program. Target LDL-C levels are < 2.5 mmol/L for patients in primary prophylaxis and < 1.8 mmol/L for patients in secondary prophylaxis. These levels can be achieved using statins, ezetimibe, PCSK9-i, and LDL apheresis. FH patients are united in a working patient organization.

#### Slovenia

In this country with a population of over 2 million people, more than 4000 FH patients can be expected. More than 50% of the considered number have been diagnosed and are included in the database. There are two University centers operating in Slovenia and a specialized network of lipid clinics. Unfortunately, no more information is available.

#### Turkey

There are approximately 168,000 FH patients in this land of 84 million people with 3159 patients already integrated into the ScreenPro FH project database. As far as the management of the treatment of patients is concerned, there is a national center in Izmir and 31 regional centers. The FH program is based on the Adult HoFH Apheresis Registry, and the creation of a functioning lipid network (which had not existed in the county until now) is being planned. The diagnosis of FH is made based on the DLCNC; treatment modalities are represented not only by statins, ezetimibe, or evolocumab (only for patients with homozygous FH) but also by LDL apheresis which is performed in 18 centers. Patients are treated to achieve target LDL-C levels of compliancy described by current European guidelines. There is also a working patient organization in the country.

#### Ukraine

In Ukraine, with a population of 43 million inhabitants, the occurrence of approximately 86,000 FH cases can be expected; 147 patients have been included in the ScreenPro FH database so far. A lipid network is being developed in the country to include a national center and four regional centers. The objective of the FH program in the country is to actively search for patients with suspected FH; FH should be diagnosed with the use of the DLCNC or Simon Broom Criteria. Patients can be treated with statins and ezetimibe to achieve the target LDL-C levels of less than 1.8 mmol/L and 2.5 mmol/L, respectively, based on the category of CV risk. LDL apheresis is not available, and no patient organization has been founded in the country yet.

#### Uzbekistan

The occurrence of potential FH patients in Uzbekistan, with a population of 31 million inhabitants, is estimated to be 62,000, though there are only 106 cases in the ScreenPro FH project database. A lipid network has been created in the country, albeit at a slow pace, with the national center in Tashkent and four regional centers. The objective of the FH program is to introduce a personalized approach to the treatment of DLP patients. In these patients, the diagnosis is established based on the DLCNC; not only is treatment available with statins, but also with LDL apheresis which can be performed at two private clinics. Target LDL-C levels are set to 1.8 mmol/L. Patients with a diagnosis of FH can also register in a patient organization under the auspices of the Republican Specialized Center of Cardiology (RSCC).

## Results

Ten of the 22 countries of the CESE region took part in the project to search for FH patients and investigated the effect of the care for these patients in specialized regional center networks on the levels of total cholesterol, triglycerides, LDL-C, and HDL-cholesterol (HDL-C). These countries included the Czech Republic, Bosnia and Herzegovina, Lithuania, Latvia, Hungary, Russia, Serbia, Slovakia, Uzbekistan, and Ukraine. During the project, levels of lipid parameters were subsequently obtained from 5585 of the 9065 monitored patients. In all countries, with the exception of Ukraine, the levels of total cholesterol, triglycerides, and LDL-C during the project were always statistically significantly lower (*p* < 0.001) than at their inclusion in the project. A substantial decrease of approximately 41.5% was noticed in LDL-C levels and by approximately 32% in total cholesterol levels (Table 1). The decrease in the triglyceride levels in the monitored countries was approximately 16% while there was almost no difference in the HDL-C levels.

**Table 1:** Effect of screening and treatment on the lipid parameter levels of FH patients in 10 selected countries

	Start of the project	Follow-up	Difference (mmol/L)	Difference (%)
Cholesterol concentration (mmol/L)	8.640	5.850	2.790	32.30%
LDL-C concentration (mmol/L)	6.220	3.640	2.580	41.52%
Triglyceride concentration (mmol/L)	1.820	1.530	0.290	15.84%
HDL-C concentration (mmol/L)	1.438	1.437	0.001	0.04%

*LDL-C* LDL-cholesterol, *HDL-C* HDL-cholesterol. Average values of lipid parameters of individual countries from the beginning of the project and after the inclusion of the patients to the national database were summed and related to the number of patients, and the difference of the given lipid parameter values was evaluated; this difference was related to the total level of lipid parameter at the start of the project

Patient representation was markedly different in each of the 10 countries (Appendix Table 6). In the Czech Republic, only 3256 patients, from the original group of 4045 patients included in the project, were continuously monitored. This situation was similar in Russia and Slovakia, where 699 patients from the original group of 1200 patients and 200 patients from the original group of 2246 patients, respectively, were continuously monitored. In Bosnia and Herzegovina, 343 patients were included in the project and continuously monitored. Similarly in Serbia, Uzbekistan, and Ukraine, the number of patients monitored were 302, 106, and 147 respectively. Small differences between the numbers of monitored patients at the project entrance in comparison with the number of patients monitored during the project were seen in Lithuania, Latvia, and Hungary. In Lithuania, there were 98 patients included in the project and this number increased up to 100 patients in the course of the study. In Latvia, there were 249 patients included in the project and 105 patients had at least one follow-up visit. In Hungary, there were 329 patients included in the study and 327 patients continued; thus, a minimum decrease of patients was observed.

**Fig. 1:**
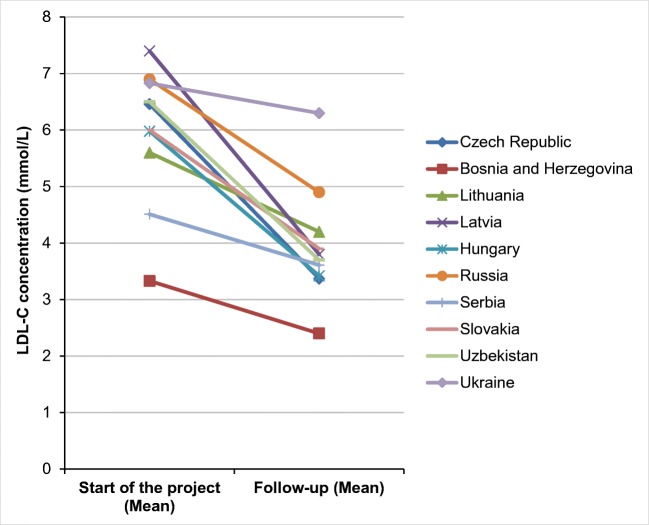
Comparison of LDL-C Levels at the project entrance and during the project in individual countries

**Fig. 2:**
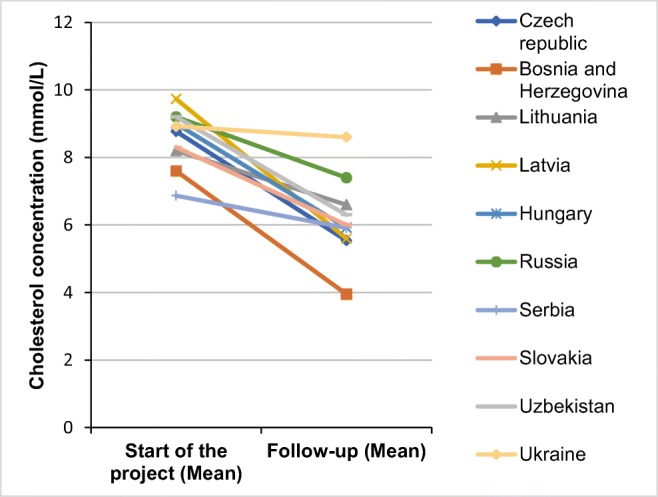
Comparison of total cholesterol levels at the project entrance and during the project in individual countries

**Fig. 3:**
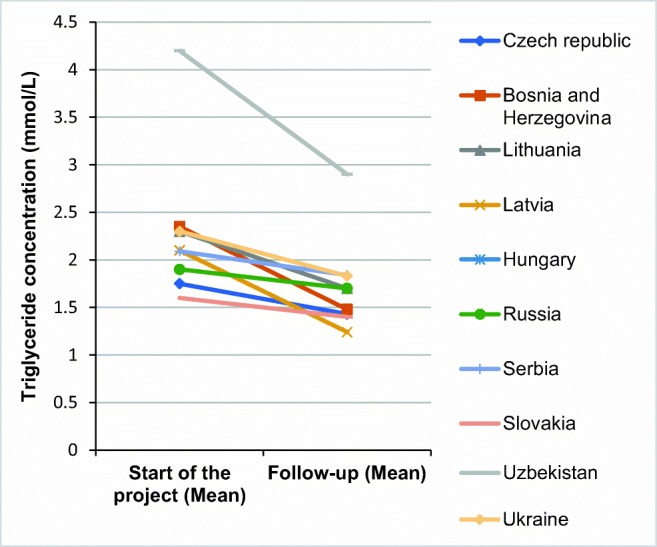
Comparison of triglyceride levels at the project entrance and during the project in individual countries

The levels of triglycerides and HDL-C were compared in only nine selected countries (Tables 2 and 3). Hungary was not included in the comparison of these two lipid parameters. However, the levels of total cholesterol and LDL-C were compared in all selected countries (Tables 4 and 5). The decreases in the levels of total cholesterol, LDL-C, and triglycerides were statistically significant in all selected countries with the exception of Ukraine (*p* < 0.001) where a statistically significant decrease was found only in the triglyceride levels (*p* < 0.001). The decreases in the levels of total cholesterol and LDL-C were not statistically significant (*p* = 0.276 and *p* = 0.068, respectively) in Ukraine, though a decrease in the values of these two lipid parameters was reported. As far as total cholesterol is concerned, a significant decrease in its levels was observed in Bosnia and Herzegovina, and Latvia (Fig. 2), in particular, where the values of total cholesterol decreased by more than 40%. Decreases in levels of total cholesterol by approximately 35% were also observed in the Czech Republic and Hungary, and by 25–30% in Slovakia and Uzbekistan. Decreases of approximately 20% were reported in Russia, Serbia, and Lithuania. A 3.6% decrease in the levels of total cholesterol was observed in Ukraine. Significant decreases in LDL-C levels were observed in the Czech Republic, Latvia, Hungary, and Uzbekistan where the levels of LDL-C decreased by more than 40% (Fig. 1). Decreases in LDL-C levels of approximately 25–35% were reported in Bosnia and Herzegovina, Lithuania, Russia, and Slovakia. Only in Serbia and Ukraine did the values of LDL-C decrease by approximately 20% and 7.8%, respectively. In Bosnia and Herzegovina as in Latvia, the triglyceride levels significantly decreased by more than 35%. In Lithuania and Uzbekistan, the triglyceride levels decreased by 26–31% (Fig. 3). Slight decreases, 18–21%, in the levels of triglycerides were observed in the Czech Republic and Ukraine, and by no more than 13% in Russia, Serbia, and Slovakia.

**Table 2:** Comparison of triglyceride values at the project entrance and during the project in individual countries

Triglyceride concentration (mmol/L)	Start of the project		Follow-up		*p* value
	Mean	SD	Mean	SD	
Czech Republic	1.75	0.81	1.43	0.83	*p* < 0.001
Bosnia and Herzegovina	2.35	1.63	1.48	0.50	*p* < 0.001
Lithuania	2.30	1.80	1.70	1.00	*p* < 0.001
Latvia	2.10	1.46	1.24	0.60	*p* < 0.001
Hungary					
Russia	1.90	1.20	1.70	1.00	*p* < 0.001
Serbia	2.09	0.99	1.84	0.82	*p* < 0.001
Slovakia	1.60	0.70	1.40	0.70	*p* < 0.001
Uzbekistan	4.20	0.70	2.90	0.80	*p* < 0.001
Ukraine	2.30	0.93	1.83	0.93	*p* < 0.001

*SD* standard deviation

**Table 3:** Comparison of HDL**-**C values at the project entrance and during the project in individual countries

HDL-C concentration (mmol/L)	Start of the project		Follow-up		*p* value
	Mean	SD	Mean	SD	
Czech Republic	1.52	0.47	1.52	0.41	*p* = 1.000
Bosnia and Herzegovina	1.03	0.33	1.00	0.22	*p* = 0.162
Lithuania	1.30	0.40	1.20	0.20	*p* = 0.027
Latvia	1.69	0.65	1.49	0.42	*p* = 0.004
Hungary					
Russia	1.40	0.50	1.40	0.40	*p* = 1.000
Serbia	1.32	0.37	1.34	0.45	*p* = 0.551
Slovakia	1.40	0.50	1.50	0.60	*p* = 0.008
Uzbekistan	1.00	0.40	1.10	0.30	*p* = 0.041
Ukraine	1.23	0.33	1.28	0.33	*p* = 0.195

*HDL****-****C* HDL**-**cholesterol, *SD* standard deviation

**Table 4:** Comparison of total cholesterol values at the project entrance and during the project in individual countries

Cholesterol concentration (mmol/L)	Start of the project		Follow-up		*p* value
	Mean	SD	Mean	SD	
Czech Republic	8.77	1.57	5.54	1.43	*p* < 0.001
Bosnia and Herzegovina	7.60	1.43	3.95	0.86	*p* < 0.001
Lithuania	8.20	2.10	6.60	1.10	*p* < 0.001
Latvia	9.73	2.70	5.60	2.04	*p* < 0.001
Hungary	9.00	2.44	5.92	2.61	*p* < 0.001
Russia	9.20	2.00	7.40	2.30	*p* < 0.001
Serbia	6.87	1.93	5.91	1.39	*p* < 0.001
Slovakia	8.30	1.40	6.00	1.60	*p* < 0.001
Uzbekistan	9.20	2.70	6.30	1.80	*p* < 0.001
Ukraine	8.92	2.58	8.60	2.43	*p* = 0.276

*SD* standard deviation

**Table 5:** Comparison of LDL-C values at the project entrance and during the project in individual countries

LDL-C concentration (mmol/L)	Start of the project		Follow-up		*p* value
	Mean	SD	Mean	SD	
Czech Republic	6.46	1.53	3.37	1.33	*p* < 0.001
Bosnia and Herzegovina	3.33	1.15	2.40	0.84	*p* < 0.001
Lithuania	5.60	2.10	4.20	1.20	*p* < 0.001
Latvia	7.19	1.86	3.53	1.78	*p* < 0.001
Hungary	5.98	2.34	3.42	2.25	*p* < 0.001
Russia	6.90	1.70	4.90	2.40	*p* < 0.001
Serbia	4.51	1.69	3.61	1.19	*p* < 0.001
Slovakia	6.00	1.40	3.90	1.50	*p* < 0.001
Uzbekistan	6.50	1.70	3.70	1.10	*p* < 0.001
Ukraine	6.83	2.48	6.30	2.48	*p* = 0.068

*LDL-C* LDL**-**cholesterol, *SD* standard deviation

**Fig. 4:**
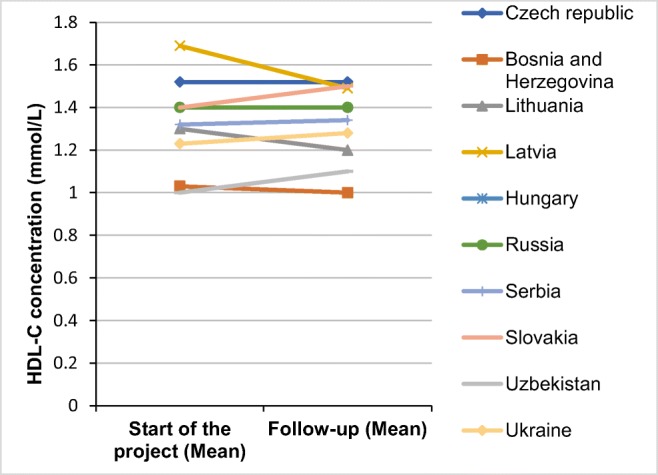
Comparison of HDL-C levels at the project entrance and during the project in individual countries

In the case of HDL-C, statistically significant lower levels, when compared with the baseline values, were found in Lithuania (from 1.3 to 1.2, *p* = 0.027) and Latvia (a decrease from 1.69 to 1.49, *p* = 0.004), and statistically significant higher levels were found in Slovakia (an increase from 1.4 to 1.5, *p* = 0.008) and Uzbekistan (an increase from 1.0 to 1.1, *p* = 0.041). In the Czech Republic, Bosnia and Herzegovina, Russia, Serbia, and Ukraine, the baseline HDL-C levels remained almost the same during the project in comparison with the baseline (Fig. 4).

## Discussion

The ScreenPro FH is not the only international activity in the field of FH. In the Pacific region, a similar project, the “Ten Countries Study,” was carried out, with similar intentions and results [37, 38]. The biggest and only true global project is the FHSC [39]. It intends to create a global database which would be optimal both for data collection and their quality. On the other hand, there are mainly legal obstacles (of all kinds) in several countries which slow the recruitment of patients. Our study uses data summarized for each country. Consequently, the data of individual patients are not, for all intents and purposes, released from home countries. This explains why our group of FH patients is one of the biggest groups when compared with the global database.

The most valuable result of our project is considered to be the significant (not only statistically but mainly clinically) change in the lipid spectrum, in particular the decrease in LDL-C levels by more than 40% and the decrease of total cholesterol levels by more than 30% in patients from their inclusion to the database to post-intervention. Thus, we consider the results undoubtedly positive, despite the fact that we were not able to include results from all patients in the databases due to the lack of “before and after” data.

However, it must be stated that our results have only limited validity. In addition, it is necessary to mention that our patients are far from the target levels of LDL-C and total cholesterol. Nonetheless, this represents the first analysis; in many centers, physicians have a great opportunity to use higher doses of lipid-lowering drugs. Also, it will soon be possible, at least in some countries, to introduce monoclonal antibodies, evolocumab, and alirocumab. Regarding the change in triglycerides, although it is positive, we do not consider it significant. The change in HDL-C, which is minimal from a clinical point of view, is not considered substantial.

So far in the project, we have not paid much attention to treatment with LDL apheresis which is available in some countries; however, it can be considered highly selective and often aimed only at FH homozygotes.

## Conclusions

As pointed out several times in the past, FH represents a significant CV risk. On the other hand, there are several treatment options: currently available standard treatment (statins + ezetimibe) and up-to-date treatment, MAB (PCSK9-i), as well as bempedoic acid [32], which is currently in development, or inclisiran. The search for patients and their early treatment is thus legitimate. The ScreenPro FH project exemplifies the benefits of the contributions of an international community to improving screening, diagnostics, and treatment of FH patients. It is further proof that sharing information, assisting in education, and increasing awareness can lead to positive changes in lipids, especially to a significant decrease in LDL-C in FH patients.

It can be generally concluded that the international cooperation in the ScreenPro FH project has led to a decrease in the CV risk in FH patients included in national databases. In further studies, we would like to focus on two issues in particular.

1. To increase the number of patients included in national databases
  - By increasing the number of cooperating centers
  - By increasing awareness in both the general population and among medical experts
  - By supporting patient organizations in individual countries
2. To improve FH patients’ comprehensive treatment and improve the effects of the treatment with lipid-lowering drugs, so that, in optimal cases, the target values are achieved

## Appendix

**Table 6:** Comparison of lipid parameter values at the project entrance and during the project in individual countries

Country	Start of the project		Follow-up		*p* value
	Mean	SD	Mean	SD	
Czech Republic	4045 patients		3256 patients		
Cholesterol concentration (mmol/L)	8.77	1.57	5.54	1.43	*p* < 0.001
LDL-C concentration (mmol/L)	6.46	1.53	3.37	1.33	*p* < 0.001
Triglyceride concentration (mmol/L)	1.75	0.81	1.43	0.83	*p* < 0.001
HDL-C concentration (mmol/L)	1.52	0.47	1.52	0.41	*p* = 1.000
Bosnia and Herzegovina	343 patients		343 patients		
Cholesterol concentration (mmol/L)	7.60	1.43	3.95	0.86	*p* < 0.001
LDL-C concentration (mmol/L)	3.33	1.15	2.40	0.84	*p* < 0.001
Triglyceride concentration (mmol/L)	2.35	1.63	1.48	0.50	*p* < 0.001
HDL-C concentration (mmol/L)	1.03	0.33	1.00	0.22	*p* = 0.162
Lithuania	98 patients		100 patients		
Cholesterol concentration (mmol/L)	8.2	2.1	6.6	1.1	*p* < 0.001
LDL-C concentration (mmol/L)	5.6	2.1	4.2	1.2	*p* < 0.001
Triglyceride concentration (mmol/L)	2.3	1.8	1.7	1.0	*p* < 0.001
HDL-C concentration (mmol/L)	1.3	0.4	1.2	0.2	*p* = 0.027
Latvia	249 patients		105 patients		
Cholesterol concentration (mmol/L)	9.73	2.70	5.60	2.04	*p* < 0.001
LDL-C concentration (mmol/L)	7.19	1.86	3.53	1.78	*p* < 0.001
Triglyceride concentration (mmol/L)	2.10	1.46	1.24	0.60	*p* < 0.001
HDL-C concentration (mmol/L)	1.69	0.65	1.49	0.42	*p* = 0.004
Hungary	329 patients		327 patients		
Cholesterol concentration (mmol/L)	9.00	2.44	5.92	2.61	*p* < 0.001
LDL-C concentration (mmol/L)	5.98	2.34	3.42	2.25	*p* < 0.001
Triglyceride concentration (mmol/L)					
HDL-C concentration (mmol/L)					
Russia	1200 patients		699 patients		
Cholesterol concentration (mmol/L)	9.2	2.0	7.4	2.3	*p* < 0.001
LDL-C concentration (mmol/L)	6.9	1.7	4.9	2.4	*p* < 0.001
Triglyceride concentration (mmol/L)	1.9	1.2	1.7	1.0	*p* < 0.001
HDL-C concentration (mmol/L)	1.4	0.5	1.4	0.4	*p* = 1.000
Serbia	302 patients		302 patients		
Cholesterol concentration (mmol/L)	6.87	1.93	5.91	1.39	*p* < 0.001
LDL-C concentration (mmol/L)	4.51	1.69	3.61	1.19	*p* < 0.001
Triglyceride concentration (mmol/L)	2.09	0.99	1.84	0.82	*p* < 0.001
HDL-C concentration (mmol/L)	1.32	0.37	1.34	0.45	*p* = 0.551
Slovakia	2246 patients		200 patients		
Cholesterol concentration (mmol/L)	8.3	1.4	6.0	1.6	*p* < 0.001
LDL-C concentration (mmol/L)	6.0	1.4	3.9	1.5	*p* < 0.001
Triglyceride concentration (mmol/L)	1.6	0.7	1.4	0.7	*p* < 0.001
HDL-C concentration (mmol/L)	1.4	0.5	1.5	0.6	*p* = 0.008
Uzbekistan	106 patients		106 patients		
Cholesterol concentration (mmol/L)	9.2	2.7	6.3	1.8	*p* < 0.001
LDL-C concentration (mmol/L)	6.5	1.7	3.7	1.1	*p* < 0.001
Triglyceride concentration (mmol/L)	4.2	0.7	2.9	0.8	*p* < 0.001
HDL-C concentration (mmol/L)	1.0	0.4	1.1	0.3	*p* = 0.041
Ukraine	147 patients		147 patients		
Cholesterol concentration (mmol/L)	8.92	2.58	8.60	2.43	*p* = 0.276
LDL-C concentration (mmol/L)	6.83	2.48	6.30	2.48	*p* = 0.068
Triglyceride concentration (mmol/L)	2.30	0.93	1.83	0.93	*p* < 0.001
HDL-C concentration (mmol/L)	1.23	0.33	1.28	0.33	*p* = 0.195

*LDL-C* LDL-cholesterol, *HDL-C* HDL-cholesterol, *SD* standard deviation
